# Ubiquitin-Conjugating Enzyme 9 Promotes Epithelial Ovarian Cancer Cell Proliferation *in Vitro*

**DOI:** 10.3390/ijms140611061

**Published:** 2013-05-24

**Authors:** Mei Dong, Xiaoyan Pang, Yang Xu, Fang Wen, Yi Zhang

**Affiliations:** Department of Gynecology, the First Affiliated Hospital of China Medical University, Shenyang 110001, Liaoning, China; E-Mails: dongmei__2006@sina.com (M.D.); sypxyf@163.com (X.P.); wangzhixuyang@126.com (Y.X.)

**Keywords:** epithelial ovarian cancer (EOC), ubiquitin-conjugating enzyme 9 (Ubc9), proliferation, Akt

## Abstract

Epithelial ovarian cancer (EOC) is one of the leading causes of cancer deaths in women worldwide. Ubiquitin-conjugating enzyme 9 (Ubc9), the sole conjugating enzyme for sumoylation, regulates protein function and plays an important role in sumoylation-mediated cellular pathways. Although sumoylation plays a key role in DNA repair and tumorgenesis, whether Ubc9 is involved in EOC progression remains unknown. In the present study, we constructed Ubc-9 expressed recombined plasmid pEGFP-N1-Ubc9. The mRNA levels of Ubc9 were confirmed in human ovarian cell lines before and after transfection with pEGFP-N1-Ubc9 or small interfering RNA (siRNA) targeted Ubc9 by real-time polymerase chain reaction (PCR). The MTT (3-(4,5-dimethylthiazol-2-yl)-2,5-diphenyltetrazolium bromide) assay was used to observe the effect of Ubc9 on cell proliferation. The protein levels of Ubc9, and proliferation-related signals Akt and physphorylated Akt were determined by Western blot. Our results showed that proliferation of EOC cells increased significantly in Ubc9 overexpressing cells, but decreased in Ubc9 knockdown cells. The physphorylation of Akt showed similar trends. In addition, the inhibitor of PI3K/Akt signaling pathway, LY294002, dramatically inhibited the growth of Ubc9 overexpressing cells. Therefore, Ubc9 gene plays an important role in cell proliferation in EOC through PI3K/Akt signaling pathway.

## 1. Introduction

Ovarian cancer is one of the most common causes of death from all cancers among women and the leading cause of death from gynecological malignancies. Epithelial ovarian cancer (EOC) is a common malignant ovarian neoplasm with poor five-year survival rate (less than 30%) without effective early diagnostic methods. Therefore, there is a critical need for better targeted therapies for ovarian cancers.

Ubiquitin-conjugating enzyme 9 (Ubc9) is an essential E2 enzyme required for small ubiquitin-related modifier (SUMO) conjugation or sumoylation [1] and it transfers the activated SUMO to different cellular protein substrates [2]. Unlike ubiquitination that normally targets proteins for degradation through proteasome pathways, sumoylation has been implicated in the regulation of protein stability, protein-protein interactions, transcriptional activity, and subcellular localization [3,4]. In addition, recent evidence indicates that Ubc9 is a multifunctional protein that can exert its functions independent of sumoylation [5–7].

Ubc9 is a single-copy gene that is frequently up-regulated in tumors. Available evidence suggests that Ubc9 is a tumor-promoting factor. Ubc9 is up-regulated in several malignancies, such as lung adenocarcinoma, colorectal cancer, prostatic cancer, breast adenocarcinoma, melanoma and ovarian carcinoma [8–12]. However, the function of Ubc9 in EOC remains poorly understood.

In the present study, we constructed Ubc-9 expressed recombined plasmid pEGFP-N1-Ubc9 and stably transfected EOC cell line overexpressing Ubc9. We found that the overexpression of Ubc9 gene can promote EOC cell proliferation.

## 2. Results and Discussion

### 2.1. Ubc9 mRNA and Protein Expression in Ovarian Cancer Cells

**Figure 1:**
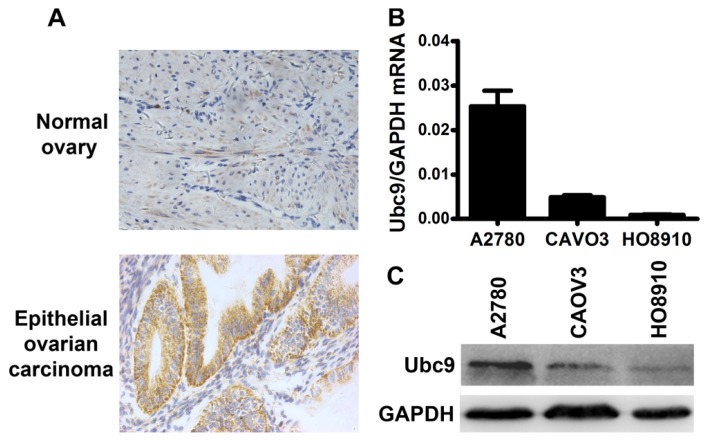
Ubc9 was expressed in ovarian epithelial carcinoma *in vivo* and *in vitro*. (**A**) Immunohistochemical analysis of sections from ovarian epithelial carcinoma samples or normal ovary samples with specific anti-Ubc9 antibody. Magnification is 400×; (**B**) Relative Ubc9 mRNA expression in epithelial ovarian carcinoma cell lines; (**C**) Ubc9 protein expression in epithelial ovarian carcinoma cell lines.

We first examined that whether Ubc9 is expressed in ovarian epithelial carcinoma tissues *in vivo*. Immunohistochemical staining showed that Ubc9 was expressed in ovarian epithelial carcinoma tissue, but barely expressed in normal ovary tissue (Figure 1A). Three cell lines were screened for Ubc9 mRNA and protein expression using real-time polymerase chain reactions (PCR) and Western blot (Figure 1B,C). Our results showed that the expression level of Ubc9 was the highest in A2780 cells and the lowest in HO-8910 cells both at mRNA and protein levels. Therefore, out of these three cell lines, HO-8910 cell was chosen for preparing Ubc9 stably overexpressing cells, because they showed relatively lower expression of Ubc9.

### 2.2. Construction of Ubc9 Recombinant Plasmid and Selection of Stable Transfected Cell Clones

**Figure 2:**
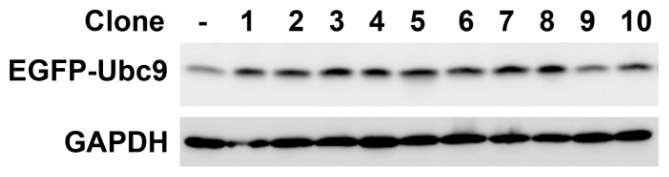
Construction of Ubc9 recombinant plasmid and selection of stable transfected cell clones. The identification of Ubc9 stably overexpressing HO-8910 cells was performed by Western Blot.

To determine the effect of Ubc9 in the proliferation of HO-8910 cells, we prepared the Ubc9 stably overexpressing cells. First, we constructed pEGFP-N1-Ubc9 recombinant plasmid successfully, the sequence was verified by DNA sequencing. Then, the pEGFP-N1-Ubc9 plasmids were transfected into HO-8910 cells to create 10 stably transfected cell lines. Each positive clone was identified by Western blot analysis for expression of EGFP-Ubc9 (Figure 2). Clone 3 was used in the following experiments.

### 2.3. Specific Silencing of the Ubc9 Gene

**Figure 3:**
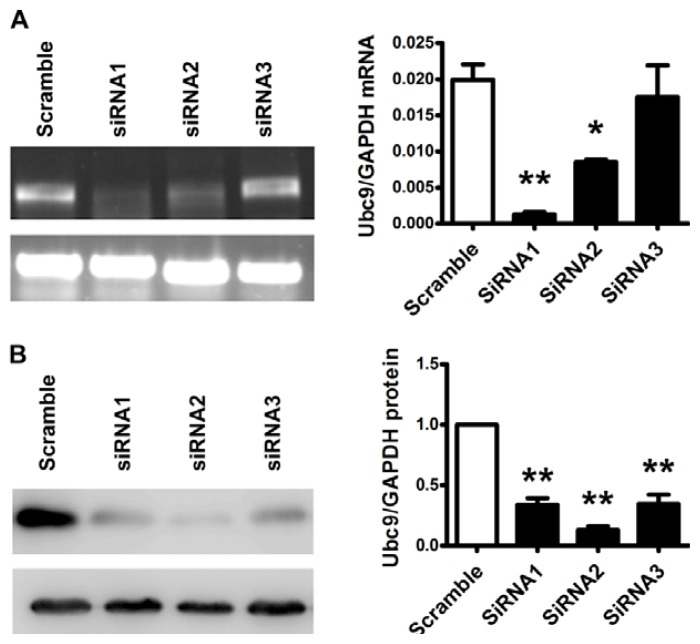
Identification of Ubc9 siRNA. (**A**) Identification of Ubc9 siRNA by real-time PCR. Relative mRNA levels were normalized to that with scramble siRNA treatment; (**B**) Ubc9 protein level in HO8910-Ubc9 cells treated with Ubc9 siRNA. The right panel represents the summary results of Ubc9 protein level. Relative protein levels were normalized to that with scramble siRNA treatment. Data are mean ± SEM from 3 separate experiments. *p < 0.05, **p < 0.01 *versus* scramble group.

Three different Ubc9 siRNA and the scrambles were transfected into HO8910-Ubc9 cells separately. The Ubc9 mRNA and protein levels were assessed by real-time PCR and western blot analysis 48 h after transfection. As shown in Figure 3A, the inhibitory rate of Ubc9 siRNA1 was 93.64% (*p* = 0.001), the inhibitory rate of Ubc9 siRNA2 was 57.32% (*p* = 0.006). However, there were no significant differences between the Ubc9 siRNA3 group and the scramble group (*p* = 0.648). By using western blot analysis as shown in Figure 3B, Ubc9 siRNA2 was found to have the highest inhibitory rate 85.28% (*p* = 0.01). Thus, we used Ubc9 siRNA1 and siRNA2 for subsequent experiments.

### 2.4. Ubc9 Overexpression Promotes Cell Proliferation, whereas siRNA Targeting Ubc9 Inhibited Epithelial Ovarian Cancer Cell Proliferation

**Figure 4:**
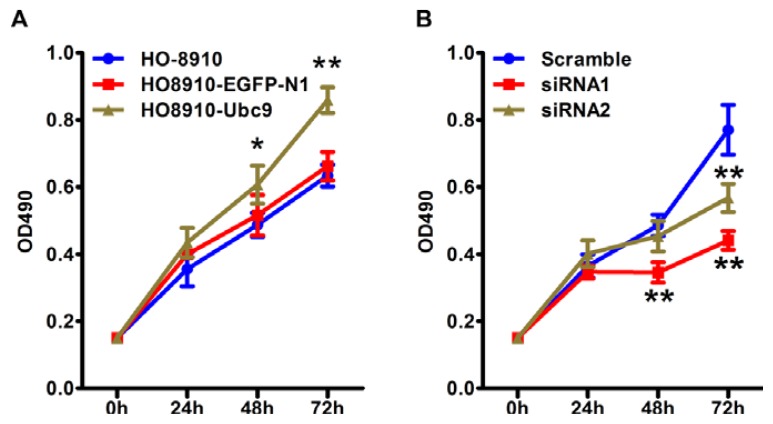
Effect of Ubc9 on HO-8910 cell proliferation. (**A**) HO-8910 cells were stably transfected with Ubc9 expressing plasmid or vectors and cell viability was detected with MTT assay. Data are means ± SEM from 3 separate experiments. *p < 0.01, **p < 0.001 compared with HO-8910 cells; (**B**) HO-8910 cells were transfected with siRNAs targeting Ubc9 or scramble siRNA and cell viability was detected with MTT assay. Data are means ± SEM from 3 separate experiments. **p < 0.001 compared with scramble transfection.

We then studied the possible role of Ubc9 in epithelial ovarian carcinoma progression. Overexpression of Ubc9 significantly increased HO-8910 cell proliferation. The proliferation rate of the HO8910-Ubc9 cells was much higher than the HO-8910 cells (*p* = 0.001, *p* < 0.001, Figure 4A) and HO8910-pEGFP-N1 vector cells at 48 and 72 h (*p* = 0.022, *p* < 0.001, Figure 4A). There was no significance difference between the HO-8910 and HO8910-pEGFP-N1 vector cells at time point 24, 48, 72 h (*p* = 0.067, *p* = 0.333, *p* = 0.226).

To further investigate the role of Ubc9 in cell proliferation, we suppressed Ubc9 expression by Ubc9 specific siRNAs. HO8910-Ubc9 cell presented relatively higher expression of Ubc9 and siRNA1 and2 were chosen for RNA interference. We measured the cell growth by MTT assay. As shown in Figure 4B, the proliferation rate was significantly inhibited by siRNA1 transfection compared with scramble group at 48, 72 h (*p* < 0.001, *p* < 0.001), and the proliferation rate of siRNA2 group was also inhibited compared with scramble group at 72 h (*p* < 0.001).

### 2.5. PI3K/Akt Signaling is Required for Ubc9 in Accelerating HO8910 Cell Proliferation

**Figure 5:**
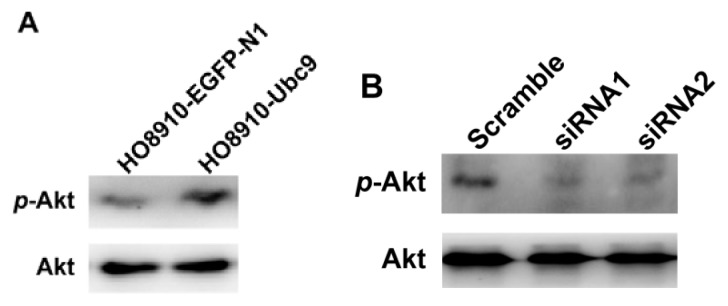

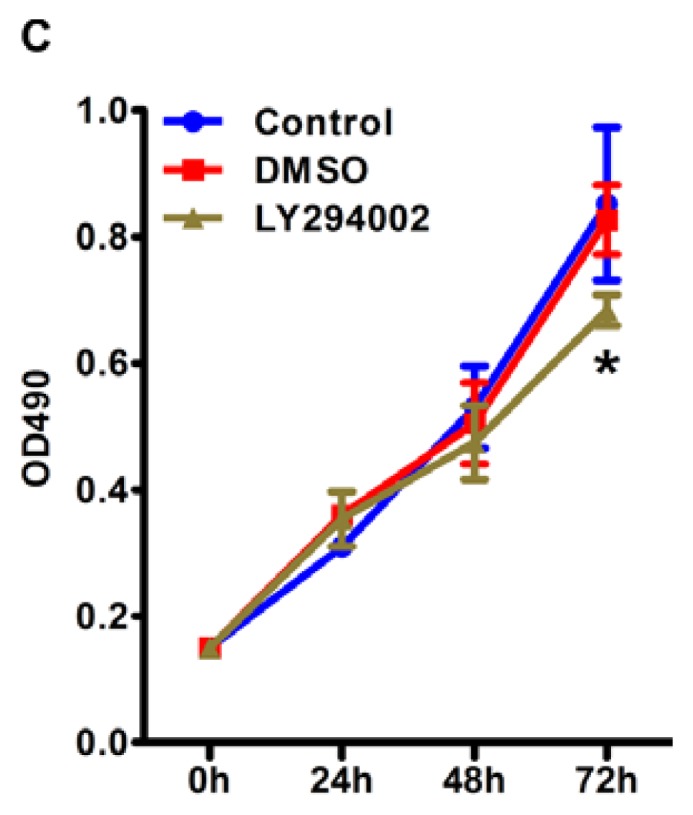
PI3K/Akt signaling is required for Ubc9 in accelerating HO-8910 cell proliferation. (**A**) Western blot profiles of Akt and p-Akt proteins in HO8910 and HO8910-Ubc9 cells; (**B**) Western blot profiles of Akt and p-Akt proteins in Scramble- and Ubc9-siRNA-transfected HO8910-Ubc9 cells; (**C**) The proliferation of HO8910-Ubc9 cells were inhibited after being treated with LY294002. Data are means ± SEM from 3 separate experiments. *p < 0.01, compared with DMSO-treated HO8910-Ubc9 cells.

In growth factor signaling, activation of Akt has been implicated as a key step. As shown in Figure 5, phosphorylation of Akt protein was apparently increased in Ubc9 overpressing cells (Figure 5A) and decreased in Ubc9-knockdown cells (Figure 5B). Therefore, we then detected the role of Akt in Ubc9-augmented EOC cell proliferation. As shown in Figure 5C, after HO8910-Ubc9 cells were treated by LY294002 (25 μM), the cell proliferation rates were much lower than untreated HO8910-Ubc9 cells (*p* = 0.038) and DMSO(dimethylsulfoxide)-treated group (*p* = 0.003) at time point 72 h. There was no significant difference between HO8910-Ubc9 and DMSO group (*p* = 0.717). These results indicated that involvement of Ubc9 in the proliferation of epithelial ovarian carcinoma was at least partially through the PI3K/Akt pathway.

### 2.6. Discussion

The present study demonstrated that Ubc9 can promote the proliferation of human epithelial ovarian carcinoma cell line HO-8910 cells at least partially through a PI3K/Akt signaling pathway. This conclusion is supported by the following observations: (1) Ubc9 is expressed in EOC tissue *in vivo*; (2) Proliferation of EOC cells increased significantly in Ubc9 overexpressing HO-8910 cells, but decreased in Ubc9 knockdown HO-8910 cells; (3) Phosphorylation of Akt increased significantly in Ubc9 overexpressing HO-8910 cells, but decreased in Ubc9 knockdown HO-8910 cells; (4) Inhibition of PI3K/Akt signaling pathway by LY294002 dramatically inhibited the growth of Ubc9 overexpressing HO-8910 cells. To the best of our knowledge, this is the first report demonstrating the involvement of Ubc9 in the proliferation of epithelial ovarian carcinoma.

Sumoylation is the covalent attachment of SUMO to a target protein. Similar to other ubiquitin-like pathways, three enzyme types are involved that act in succession: an activating enzyme (E1), a conjugating enzyme (E2), and a ligase (E3). Protein sumoylation has been implicated in the regulation of protein stability, subcellular localization, and the activity of transcription factors [13,14]. Because Ubc9 is the sole E2-conjugating enzyme required for sumoylation, it is believed to play a central role in these processes. Ubc9 is well known for its key role in protein sumoylation and sumoylation-mediated cellular pathways, ultimately impacting tumor initiation and progression [12,15]. Dysregulation of Ubc9 may lead to alterations in the sumoylation process or may only affect the sumoylation modification of some key proteins, ultimately impacting cell growth and cancer development.

As a multi-functional protein, some evidence suggests that Ubc9 can also promote breast cell invasion and metastasis in a sumoylation independent manner [8]. However, there is scarce information available in the literature as to whether and how Ubc9 impacts on ovarian cancer cells, although previous studies have reported that Ubc9 was up-regulated in ovarian cancer tissues and cells [12,16]. In this study, we introduced Ubc9 gene into human ovarian cancer cell line HO-8910 cells through gene transfection and established cell model overexpressing Ubc9 gene stably. We found that Ubc9 can promote cell proliferation in EOC, which may give the potential target for EOC therapy in the future.

PI3K/Akt signaling pathway is a critical pathway involved in cell survival and has been shown to be constitutively active in ovarian cancer cell lines [17]. We found that the phosporylation of Akt was increased in Ubc9 overpressing HO-8910 cells, but knockdown of endogenous Ubc9 resulted in suppression Akt phosporylation. Furthermore, Ubc9 related proliferation of HO-8910 cells can be inhibited by the inhibitor of a PI3K/Akt signaling pathway, LY294002. Hence, we infer that PI3K/Akt signaling is required for Ubc9 in accelerating HO8910 cell proliferation. A number of studies have demonstrated that the patients with activated Akt had a significant survival disadvantage compared to patients with lower level of Akt phosphorylation, and the patients with ovarian cancer suggested Akt phosphorylation as an independent prognostic indicator [16,18,19]. To our knowledge, this is the first report showing that overexpression of Ubc9 could significantly enhance proliferation of ovarian cancer cells through the PI3K/Akt pathway.

In summary, this study showed a novel connection among Ubc9, cell proliferation and epithelial ovarian cancer. Additional studies are needed to better define how these interactions are initiated and regulated, and to demonstrate whether similar effects play a role *in vivo*.

## 3. Experimental Section

### 3.1. Reagents

Mouse-anti-human Ubc9 (sc-271057), mouse-anti-human GAPDH (sc-365062), goat-anti-mouse (sc-2005) and goat-anti-rabbit (sc-2005) secondary antibodies were purchased from Santa Cruz (Santa Cruz, CA, USA). Primary polyclonal Rabbit-anti-total (No. 4685) and phosphorylated (No. 4058) Akt antibodies were from Cell Signaling Technology Inc. (Beverly, MA, USA). Expression vector pEGFP-N1, RIPM-1640 and Dulbecco’s modified Eagle’s medium (DMEM), fetal bovine serum (FBS), Trizol reagent, Lipofectamine 2000, restriction endonucleases BamHI, XhoI and G418 (geneticin) were from Invitrogen (San Diego, CA, USA). *E. coli* (competent cells) JM109 were from Toyo (Tokyo, Japan). PCR primers and agarose gel were from Sangon Biotech (Shanghai China). Reverse Transcription Polymerase Chain Reaction (RT-PCR) kit and reagents, SYBR Ex Script™ RT-PCR Kit were from Takara (Dalian, China). siRNAs targeted Ubc9 and scrambles were from Genkan (Shanghai, China). Ethylenediamine tetraacetic acid (EDTA), dimethyl sulfoxide (DMSO) and 3-(4,5-dimethylthiazol-2-yl)-2,5-diphenyltetrazolium bromide (MTT) were from Amresco (Solon, OH, USA). PI3K inhibitor LY294002 was from Promega (Madison, WI, USA). BCA Protein Assay Kit and ECL kit were from Pierce (Rockford, IL, USA).

### 3.2. Cell Culture

Three human ovarian cell lines were used in this study. HO8910 and CAOV3 cells were grown in DMEM and A2780 cells were grown in RPMI-1640 medium. Both were supplemented with 10% FBS and maintained continuously in a humidified, 5% CO_2_ incubator at 37 °C.

### 3.3. Immunohistochemical Analysis

Frozen sections of ovarian samples were incubated with mouse primary anti-Ubc9 antibody or purified mouse IgG, horseradish peroxidase-conjugated goat anti-mouse IgG and 3, 3-diaminobenzidine successively. Sections were then counterstained with hematoxylin.

### 3.4. Quantitative Real-Time PCR

Total RNA from cells were extracted using Trizol (Invitrogen, Carlsbad, CA, USA) according to the manufacturer’s instructions and 2 μg of each total RNA sample was aliquoted to synthesize cDNA using the Reverse Transcription System (TaKaRa, Tokyo, Japan). The real-time PCR was run using in a 25 μL reaction volume on 96-well plates by using the SYBR Ex Script™ RT-PCR Kit (TaKaRa, Tokyo, Japan) according to the manufacturer’s instructions on the ABI Prism 7,500 Detection System (Applied Biosystems, Foster City, CA, USA). The primers for human Ubc9 were F: 5′-GTC CTC CAC CTG TCC GCT AC-3′, R: 5′-TCT TGC CAA ACC AAT CCC T-3′ (PCR product size, 476 bp); the primers for human GAPDH were F: 5′-GAA GGT GAA GGT CGG AGT C-3′, R: 5′-GAA GAT GGT GAT GGG ATT TC-3′ (PCR product size, 230 bp). All reactions were done in duplicate and all experiments were repeated at least three times. Levels of mRNA were quantified based on the ratio of Ubc9 mRNA/GAPDH mRNA using the 2-ΔΔ*CT* method as described by the manufacturer (User Bulletin #2, Applied Biosystems, Foster City, CA, USA).

### 3.5. Western Blot

Following treatment, the cells were packed by centrifuging the cells for 3 min at 200 × *g*, and homogenized in ice-cold fractionation buffer (50 mM Tris-HCl, pH 7.4, 1 mM EDTA, 150 mM NaCl, 1% Triton X-100, 1 mM PMSF, 10 μg/mL leupeptin, 10 μg/mL pepstatin A, 10 μg/mL aprotinin, 1 mM sodium orthovanadate (Na_3_VO_4_), 10 mM sodium pyrophosphate (Na_4_P_2_O_7_) and 50 mM sodium fluoride (NaF)). The cell lysate was incubated on ice for 15 min and then centrifuged at 20,000 × *g* for 30 min at 4 °C. The cytosolic fraction was collected and subjected to SDS-PAGE with a 10% running gel. Protein concentrations were determined by the BCA(Bicinchoninic Acid) Protein Assay Kit. The proteins were transferred to a polyvinylidene fluoride membrane. The membrane was incubated successively with 5% bovine serum albumin in tris tween buffered saline (TTBS) at room temperature for 1 h, with different first antibodies at 4 °C for 12 h, and then with horseradish peroxidase-labeled second antibody for 1 h. After each incubation, the membrane was washed extensively with TTBS, and the immunoreactive band was detected with ECL-detecting reagents.

### 3.6. Construction of Plasmid and Generation of Stably Transfected Cell Lines

The Ubc9 gene was amplified by PCR with human leukocyte genomic DNA as a template and primers according to the Ubc9 gene sequence (Gene Bank Accession Number: M003345), sense primer, 5′-GTC CTC CAC CTG TCC GC TA C-3′, and antisense primer, 5′-TCT TGC CAA ACC AAT CCC T-3′, under the following conditions: denaturation at 94 °C for 9 min, followed by 30 cycles of 94 °C for 1 min, 58 °C for 30 s and 72 °C for 40 s, then extension at 72 °C for 10 min. The PCR products were ligated into the pUCM-T vector to clone Ubc9 gene, and the DNA sequence was determined by BigDye terminator cycle sequencing ready reaction kit and a DNA sequencer (ABI Genetic Analyzer; Perkin-Elmer/Applied Biosystems, Foster City, CA, USA). Then the Ubc9 gene in pUCM-T was cut out by double digestion with restriction enzymes, XhoI and BamHI, and ligated into the XhoI and BamHI sites of the pEGFP-N1 vector to yield pEGFP-N1-Ubc9. HO8910 transfected with plasmid containing pEGFP-N1-Ubc9, empty vector only, or medium were served as experimental, vehicle control, and blank control groups, respectively. Cells were transfected by vectors with Lipofectamine™ 2000 (Invitrogen, Carlsbad, CA, USA) following the manufacturer’s protocol. In brief, normal growth media were replaced by serum-free medium when cells were seeded at 70% confluence in a 6-well plate. Transfection was performed using 2 μg (1 μg/μL) DNA and 5 μL of Lipofectamine™ 2000 Reagent (Invitrogen, Carlsbad, CA, USA). The serum-free media were replaced by normal growth media after 16 h of transfection. After 48 h of transfection, HO8910 cells expressing EGFP-Ubc9 were selected in the presence of G418 (0.4 mg/mL). Then, several stable transfected cells were cloned. Each clone was identified by fluorescence microscopy and Western blot analysis for expression of Ubc9 (or Ubc9 fusions).

### 3.7. siRNA Treatment

Three siRNA Oligos targeting Ubc9 (Ubc9-siRNA) and negtive control (scrambled siRNA) were purchased from Genkan (Shanghai, China). The scrambled siRNA were used as no significant controls (NOS). The two complementary oligonucleotides for the three siRNA are as following: siRNA1-Ubc9, sense 5′-CAA CGA GGU AUG UUA CUG ATT-3′ and antisense 5′-GUU GCU CCA UAC AAU GAC UTT-3′; siRNA2-Ubc9, sense 5′-CGU CGC UGG AAC UCC GUA GTT-3′ and antisense 5′-GCA GCG ACC UUG AGG CAU CTT-3′; siRNA3-Ubc9, sense 5′-AGA UUU CAA CGA GGU AUG UTT-3′ and antisense 5′-GCA GCG ACC UUG AGU CAU CTT-3′. The plasmids were transfected into HO8910-Ubc9 cells using Lipofectamine™ 2000 transfection reagent (Invitrogen, Carlsbad, CA, USA) according to the manufacturers’ instructions.

### 3.8. Cell Proliferation Assay

HO8910-Ubc9 and HO8910-pEGFP-N1 cells (4 × 10^3^ cells per well) were plated into 96-well plates, HO8910 cells without any treatment were used as controls. HO8910-Ubc9 (4 × 10^3^ cells per well) were plated into 96-well plates in triplicate prior to Ubc9-siRNAs or scrambled siRNA for 48 h respectively and then incubated. Cell proliferation was assessed at time point of 0, 24, 48, 72 h using MTT assay according to the manufacturer’s instructions. In brief, MTT was added to the culture medium to yield a final MTT concentration of 0.5 mg/mL and the incubation was continued for 4 h at 37 °C. The cell lysates were dissolved with DMSO (150 μL/well) at room temperature for 10 min. Results were obtained by measuring the absorbance at a wavelength of 490 nm. The test was repeated three times.

### 3.9. Statistical Analysis

Quantitative data are presented as the means ± SEM determined from the indicated number of experiments. Statistical analysis was based on Student’s t-test for comparison of two groups or one-way ANOVA for multiple comparisons. *p* < 0.05 was used to determine statistical significance.

## 4. Conclusions

In summary, the present study demonstrated that Ubc9 gene plays an important role in cell proliferation of EOC at least partially through PI3K/Akt signaling pathway.
